# Coexistence of Vulvar Dowling-Degos Disease and Seborrhoeic Keratosis

**DOI:** 10.1155/2011/605841

**Published:** 2011-09-14

**Authors:** R. Guedes, Luiz Leite

**Affiliations:** ^1^Serviço de Dermatología, Centro Hospitalar de Vila Nova de Gaia, Rua Conceição Fernandes, 4434-502 Vila Nova de Gaia, Portugal; ^2^Departamento de Dermatología, Clínica Laser Belém, Calçada da Ajuda 72, 1300 012 Lisboa, Portugal

## Abstract

Dowling-Degos disease is a rare autosomal dominant inherited pigmentary disorder, mostly confined to the flexures. Diagnosis is established based on the clinical and histopathological correlation. The authors describe the clinical case of a female patient with vulvar involvement and multiple seborrhoeic keratoses on her face, neck, and upper trunk. Major and minor clinical manifestations of Dowling-Degos disease are discussed, with particular emphasis on the genital location of the lesions, which is a rare finding. Also the presence of seborrhoeic keratosis is discussed as a coincidence or a true-associated phenomenon.

## 1. Introduction

Dowling-Degos disease (DDD) or reticulate pigmented anomaly of the flexures is a rare autosomal dominant inherited disease [1]. The disorder usually appears after puberty and is more frequent in the female gender. Diagnosis is established based on the clinical and histopathological correlation. Until now there are approximately 50 cases described in the literature [2].

## 2. Case Report

**Figure 1:**
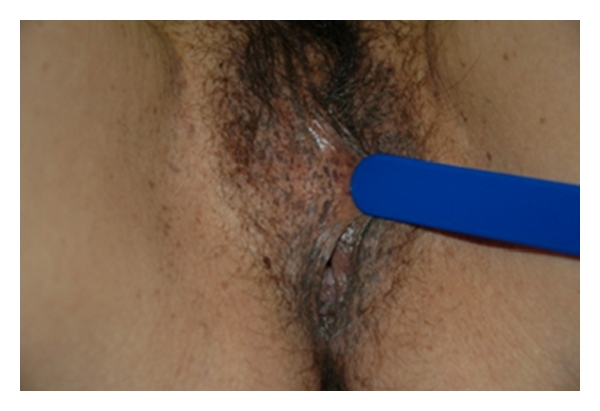
Dark-brown macules in the labia major.

**Figure 2:**
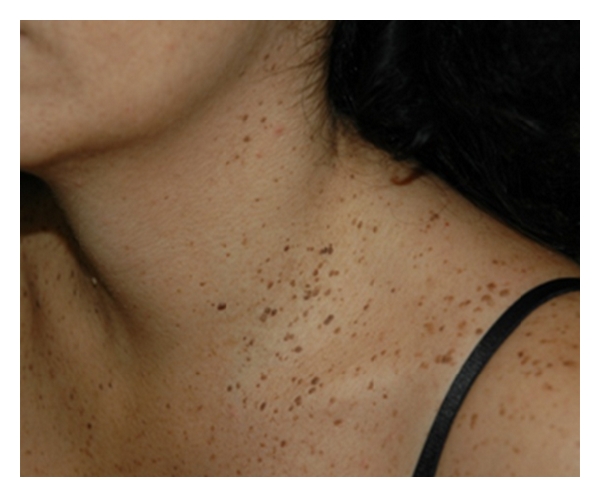
Multiple seborrhoeic keratosis over the patients' neck and upper trunk.

We describe the clinical case of a 38-year-old female patient who presented to the outpatient clinic with numerous unaesthetic, pruritic lesions on her face, neck, and upper trunk. She reported to have these lesions for more than 10 years. A few years ago she had also noticed similar lesions on her genitals. Clinically we observed multiple seborrhoeic keratosis over her face, neck, and upper trunk (Figure 1). Moreover, on the complete dermatological examination, we additionally observed multiple genital small, round, dark-brown macules with 1 mm to 0,5 cm of diameter, smooth surface, and dark-brown colour (Figure 2). The lesions had symmetrical distribution in the labia major and were asymptomatic. None of the other family members had similar findings, and she was otherwise healthy.

We performed two biopsies, namely, of one neck and one genital lesion.

**Figure 3:**
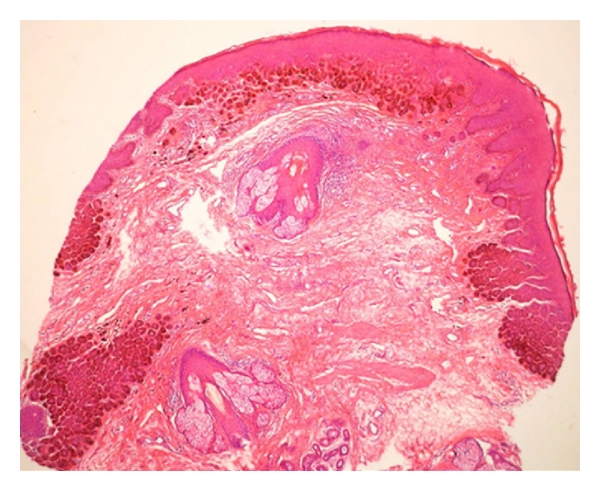
Histology findings of one vulvar lesion showing the typical elongation of the rete ridges with basal hyperpigmentation; H:E 40*.

Histology of a neck lesion revealed acanthosis, hyperkeratosis, and papillomatosis consistent with the clinical diagnosis of seborrhoeic keratosis. Histological examination of one genital macule showed elongation of rete ridges in a branching form with basal hyperpigmentation. There were no atypical keratinocytes or melanocytes (Figure 3).

These clinical and histopathological findings were consistent with the diagnosis of DDD. The patient was submitted to several carbon dioxide laser (CO_2_) sessions in order to treat the seborrhoeic keratosis. Because the genital lesions were asymptomatic, we did not perform any treatment.

## 3. Discussion

Dowling-Degos disease was described by Wilson-Jones and Grice in 1978 [3] as a reticulate pigmented anomaly of the flexures. Although some authors have proposed a loss-of-function mutation on chromosome 12 (in the KRT5 gene encoding for keratin 5), leading to melanosome uptake deficiencies [1], the exact pathophysiology is still unknown.

Clinically it is characterized by acquired hyperpigmentation affecting the flexures, pitted perioral acneiform scars, and hyperkeratotic comedo-like lesions on the neck [4]. In addition to these major features, minor features have also been described, including dystrophic fingernails, multiple keratoacanthomas, pilonidal sinus, seborrhoeic keratosis, and hidradenitis suppurativa [4].

As differential diagnosis one has to exclude acanthosis *nigricans*, Galli-Galli disease, Kitamura reticulate acropigmentation, and Haber's syndrome. Acanthosis *nigricans *is characterized by velvetlike plaques with no other minor criteria of DDD. Histopathologically, unlike DDD, papillomatosis can be observed together with the elongation of the rete ridges. Galli-Galli disease is a rare variant of DDD with acantholysis on the histology [5], and Kitamura reticulate acropigmentation affects the extensor surfaces of the hands and feet, both characteristics not observed in the described patient. Finally, Haber's syndrome was excluded due to the lack of persistent rosacea-like lesions, characteristic of this syndrome.

The association of DDD and seborrhoeic keratosis has been previously reported, and their simultaneous occurrence is based on the theory of a single underlying defect in follicular keratinization as main pathological mechanism [4]. Their occurrence, especially in young patients [6, 7], helped to establish them as a minor criterion of DDD. Likewise, genital involvement is rare, and, until today, only six case reports of DDD affecting the vulva are described in the literature [8–11], with skin lesions usually confined to the external genital areas. 

Our case is unusual in that there was reticulate hyperpigmentation affecting the vulva but without other characteristic findings (perioral scars and comedo-like lesions). Instead, multiple seborrhoeic keratoses were observed. Both are rare findings in this disorder.

## 4. Conclusion

The aim of this paper is to report the rarity of vulvar involvement in this disease and the association between seborrhoeic keratosis and DDD. In fact, seborrhoeic keratoses are not major features in this disease, but in our case they were the triggering factor for the clinical suspicion. Indeed, it is uncommon to observe patients in the 3rd decade of life with a high number of these lesions. We hope that in the future there will be more awareness of this association, in order to perform the correct diagnosis.

To our knowledge this is the first report of vulvar DDD associated with seborrhoeic keratosis.

##  Conflict of Interests

The authors declare that there is no conflict of interests.
